# Supplementary material for: Blink-induced changes in pupil dynamics are consistent and heritable
Source: Sci Rep. 2024 Nov 18;14:28421. doi: 10.1038/s41598-024-79527-4 (PMC11574171; doi:10.1038/s41598-024-79527-4)
Supplement: Supplementary file 1 — Supplementary Material 1 [file 41598_2024_79527_MOESM1_ESM.docx]

**Supplementary Tables**

**Supplementary Table 1.** Twin Demographics

Twin pairs in the final participant set of 148 participants with total of 560 runs

| **TWINS** | **Total number of pairs** | **Race** | **Gender** | **Age**  **mean(std)** |
| --- | --- | --- | --- | --- |
| **MZ** | 39 pairs | 30 White, 4 Black, 5 others | 14 Male, 25 Female pairs | 29.75 (3.61) |
| **DZ** | 32 pairs | 29 White, 2 Black, 1 other | 15 Male, 17 Female pairs  (all pairs were in the same gender) | 28.78 (3.36) |

**Supplementary Table 2.** Variability of the eye measures

| **Variable** | **Vigilant** | | | **All Drowsy** | | |
| --- | --- | --- | --- | --- | --- | --- |
|  | **Mean** | **Std** | **cv** | **Mean** | **Std** | **cv** |
| **Blinks per minute** | 17.38 | 13.84 | 0.80 | 28.31 | 18.17 | 0.64 |
| **Blink duration** | 191.92 | 48.61 | 0.25 | 202.13 | 37.69 | 0.19 |
| **Pupil size** | 722.69 | 189.37 | 0.26 | 692.05 | 197.67 | 0.29 |
| **D-peak time** | 462.40 | 89.11 | 0.19 | 497.54 | 100.25 | 0.20 |
| **D-peak amplitude** | 18.45 | 32.25 | 1.75 | 28.81 | 36.38 | 1.26 |
| **C-peak time** | 895.02 | 185.86 | 0.21 | 940.98 | 224.14 | 0.24 |
| **C-peak amplitude** | -52.21 | 37.41 | -0.72 | -41.93 | 34.40 | -0.82 |
| **D - C peak time dif.** | 440.24 | 168.40 | 0.38 | 499.56 | 252.43 | 0.51 |
| **D – C peakdrop mag.** | 69.16 | 35.15 | 0.51 | 67.77 | 37.72 | 0.56 |

**Supplementary Table 3.** Within participant correlation values for each participant within a state. Each row represents separate participants per state (just ordered as first come first locate basis).

| Vigilant | All Drowsy |
| --- | --- |
| 0.66 | 0.57 |
| 0.70 | 0.92 |
| 0.98 | 0.34 |
| 0.64 | 0.25 |
| 0.89 | 0.00 |
| 0.00 | 0.73 |
| 0.91 | 0.30 |
| 0.90 | 0.62 |
| 0.91 | 0.44 |
| 0.77 | 0.56 |
| 0.79 | 0.00 |
| 0.93 | 0.08 |
| 0.98 | 0.75 |
| 0.00 | 0.96 |
| 0.60 | 0.73 |
| 0.63 | 0.00 |
| 0.86 | 0.37 |
| 0.91 | 0.73 |
| 0.84 | 0.88 |
| 0.97 | 0.87 |
| 0.91 | 0.82 |
| 0.87 | 0.86 |
| 0.93 | 0.38 |
| 0.68 | 0.81 |
| 0.75 | 0.76 |
| 0.98 | 0.98 |
| 0.43 | 0.99 |
| 0.90 | 0.96 |
| 0.00 | 0.95 |
| 0.93 | 0.81 |
| 0.97 | 0.97 |
| 0.59 | 0.82 |
| 0.88 | 0.79 |
| 0.85 | 0.30 |
| 0.20 | 0.40 |
| 0.41 | 0.27 |
| 0.84 | 0.96 |
| 0.88 | 0.92 |
| 0.92 | 0.35 |
| 0.79 | 0.66 |
| 0.88 | 0.91 |
| 0.84 | 0.72 |
| 0.90 | 0.00 |
| 0.93 | 0.81 |
| 0.81 | 0.69 |
| 0.56 | 0.63 |
| 0.65 |  |
| 0.63 |  |
| 0.89 |  |
| 0.89 |  |
| 0.91 |  |
| 0.53 |  |
| 0.85 |  |
| 0.93 |  |
| 0.87 |  |
| 0.93 |  |
| 0.83 |  |
| 0.63 |  |
| 0.65 |  |
| 0.31 |  |
| 0.96 |  |
| 0.95 |  |
| 0.87 |  |
| 0.90 |  |
| 0.69 |  |
| 0.72 |  |
| 0.93 |  |
| 0.06 |  |
| 0.87 |  |
| 0.69 |  |
| 0.61 |  |
| 0.85 |  |
| 0.97 |  |
| 0.98 |  |
| 0.52 |  |
| 0.33 |  |
| 0.86 |  |
| 0.17 |  |
| 0.96 |  |
| 0.94 |  |
| 0.54 |  |
| 0.70 |  |
| 0.96 |  |
| 0.96 |  |
| 0.80 |  |
| 0.69 |  |

**Supplementary Table 4.** Mx model summaries and comparisons

| **BLINK PER MIN EO** | | | | | | | | | | | |
| --- | --- | --- | --- | --- | --- | --- | --- | --- | --- | --- | --- |
| **base** | **comparison** | **ep** | **minus2LL** | **df** | **AIC** | **diffLL** | **diffdf** | **p** | **fit** | **fitUnits** | **diffFit** |
| **ADE** | **NA** | **5** | **1253.0132** | **150** | **1263.013** | **NA** | **NA** | **NA** | **1253.01** | **-2lnL** | **NA** |
| **ADE** | **AE** | **5** | **1253.0158** | **150** | **1263.016** | **0.0025938** | **0** | **NA** | **1253.02** | **-2lnL** | **0.0025938** |
| ADE | E | 4 | 1265.3095 | 151 | 1273.310 | 12.296296 | 1 | 0.000454 | 1265.31 | -2lnL | 12.296296 |
| **BLINK DURATION** | | | | | | | | | | | |
| **base** | **comparison** | **ep** | **minus2LL** | **df** | **AIC** | **diffLL** | **diffdf** | **p** | **fit** | **fitUnits** | **diffFit** |
| **ADE** | **NA** | **5** | **1567.2311** | **150** | **1577.231** | **NA** | **NA** | **NA** | **1567.23** | **-2lnL** | **NA** |
| **ADE** | **AE** | **5** | **1567.2313** | **150** | **1577.231** | **0.0001388** | **0** | **NA** | **1567.23** | **-2lnL** | **0.0001388** |
| ADE | E | 4 | 1577.7975 | 151 | 1585.797 | 10.566358 | 1 | 0.001152 | 1577.80 | -2lnL | 10.566358 |
| **PUPIL SIZE** | | | | | | | | | | | |
| **base** | **comparison** | **ep** | **minus2LL** | **df** | **AIC** | **diffLL** | **diffdf** | **p** | **fit** | **fitUnits** | **diffFit** |
| ACE | NA | 6 | 2039.8810 | 151 | 2051.881 | NA | NA | NA | 2039.88 | -2lnL | NA |
| ACE | CE | 6 | 2040.1233 | 151 | 2052.123 | 0.2422733 | 0 | NA | 2040.12 | -2lnL | 0.2422733 |
| **ACE** | **AE** | **6** | **2040.1057** | **151** | **2052.106** | **0.2246134** | **0** | **NA** | **2040.11** | **-2lnL** | **0.2246134** |
| **D-PEAK TIME** | | | | | | | | | | | |
| **base** | **comparison** | **ep** | **minus2LL** | **df** | **AIC** | **diffLL** | **diffdf** | **p** | **fit** | **fitUnits** | **diffFit** |
| ACE | NA | 6 | 1112.4547 | 90 | 1124.455 | NA | NA | NA | 1112.45 | -2lnL | NA |
| ACE | CE | 6 | 1112.4924 | 90 | 1124.492 | 0.037712 | 0 | NA | 1112.49 | -2lnL | 0.037712 |
| **ACE** | **AE** | **6** | **1112.4547** | **90** | **1124.455** | **-3.46E-11** | **0** | **NA** | **1112.45** | **-2lnL** | **-3.46E-11** |
| **C-PEAK TIME** | | | | | | | | | | | |
| **base** | **comparison** | **ep** | **minus2LL** | **df** | **AIC** | **diffLL** | **diffdf** | **p** | **fit** | **fitUnits** | **diffFit** |
| **ADE** | **NA** | **5** | **1247.5222** | **91** | **1257.522** | **NA** | **NA** | **NA** | **1247.52** | **-2lnL** | **NA** |
| **ADE** | **AE** | **5** | **1247.5222** | **91** | **1257.522** | **7.39E-06** | **0** | **NA** | **1247.52** | **-2lnL** | **7.39E-06** |
| ADE | E | 4 | 1251.2289 | 92 | 1259.229 | 3.706716 | 1 | 0.054194 | 1251.23 | -2lnL | 3.706716 |
| **C-PEAK AMPLITUDE** | | | | | | | | | | | |
| **base** | **comparison** | **ep** | **minus2LL** | **df** | **AIC** | **diffLL** | **diffdf** | **p** | **fit** | **fitUnits** | **diffFit** |
| ACE | NA | 6 | 1040.4563 | 102 | 1052.456 | NA | NA | NA | 1040.46 | -2lnL | NA |
| **ACE** | **CE** | **5** | **1040.5782** | **103** | **1050.578** | **0.1219049** | **1** | **0.726978** | **1040.58** | **-2lnL** | **0.1219049** |
| ACE | AE | 5 | 1041.5298 | 103 | 1051.530 | 1.0734538 | 1 | 0.300167 | 1041.53 | -2lnL | 1.0734538 |
| **C - D PEAK TIME DIFF** | | | | | | | | | | | |
| **base** | **comparison** | **ep** | **minus2LL** | **df** | **AIC** | **diffLL** | **diffdf** | **p** | **fit** | **fitUnits** | **diffFit** |
| **ADE** | **NA** | **5** | **1196.8845** | **86** | **1206.884** | **NA** | **NA** | **NA** | **1196.88** | **-2lnL** | **NA** |
| **ADE** | **AE** | **5** | **1196.8845** | **86** | **1206.884** | **1.79E-05** | **0** | **NA** | **1196.88** | **-2lnL** | **1.79E-05** |
| ADE | E | 4 | 1201.2428 | 87 | 1209.243 | 4.3582984 | 1 | 0.036829 | 1201.24 | -2lnL | 4.3582984 |
| **D - C PEAKDROP** | | | | | | | | | | | |
| **base** | **comparison** | **ep** | **minus2LL** | **df** | **AIC** | **diffLL** | **diffdf** | **p** | **fit** | **fitUnits** | **diffFit** |
| ADE | NA | 5 | 946.3636 | 90 | 956.364 | NA | NA | NA | 946.36 | -2lnL | NA |
| ADE | AE | 5 | 946.3634 | 90 | 956.363 | -0.000252 | 0 | NA | 946.36 | -2lnL | -0.000252 |
| **ADE** | **E** | **4** | **946.4145** | **91** | **954.414** | **0.0508478** | **1** | **0.821595** | **946.41** | **-2lnL** | **0.0508478** |

**Supplementary Figures**

**Supplementary Figure 1.** Histogram of the runs per state and Framewise displacement (FD) during the MRI scan in each state


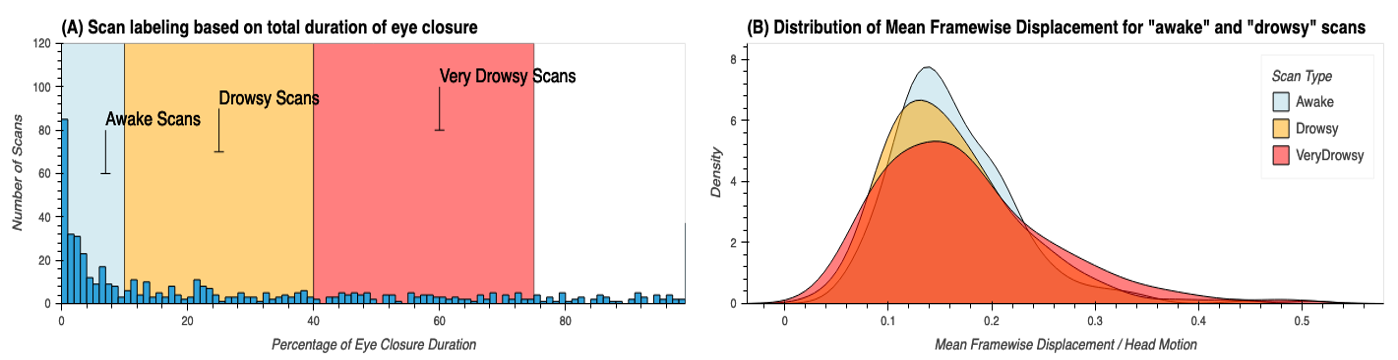


**Supplementary Figure 2.** Eye closures increase during the MRI scan particularly in the drowsy states
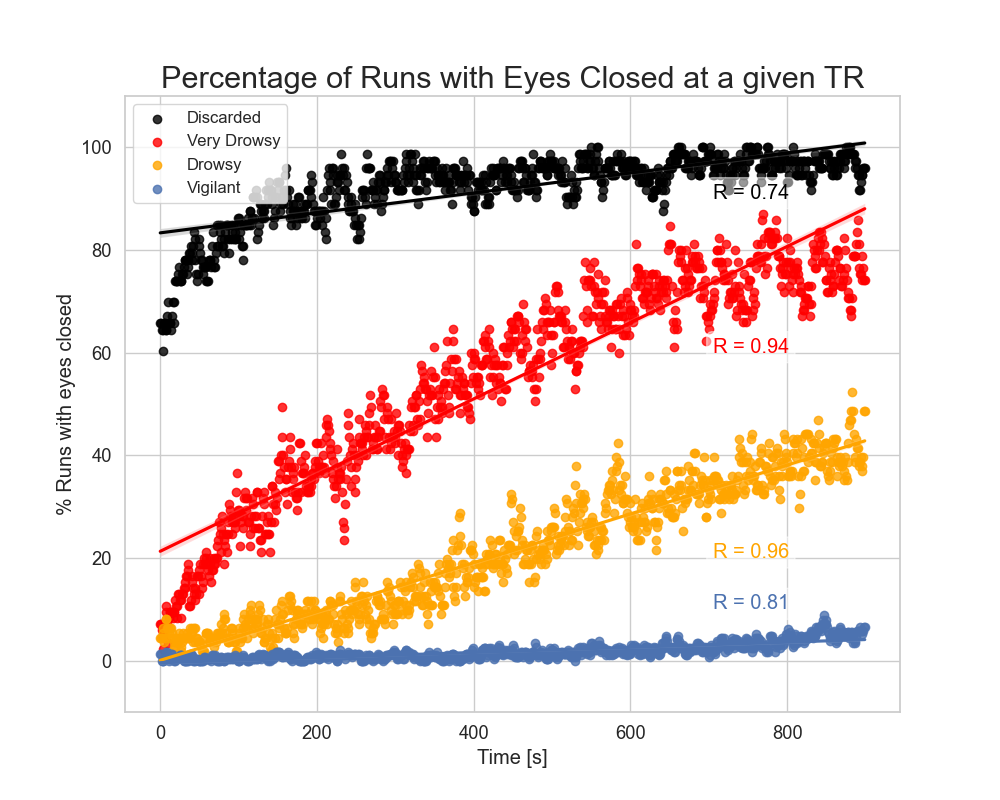


**Supplementary Figure 3.** Figure 2 extended with FD plotted on top. Blink moments marked with gray vertical lines and the eye closures marked with magenta color small vertical lines at the bottom of the figures. We also set the y-axis limits to be consistent across the vigilance states. Framewise Displacement is shown as black line at y=500 level and amplified 200 times.


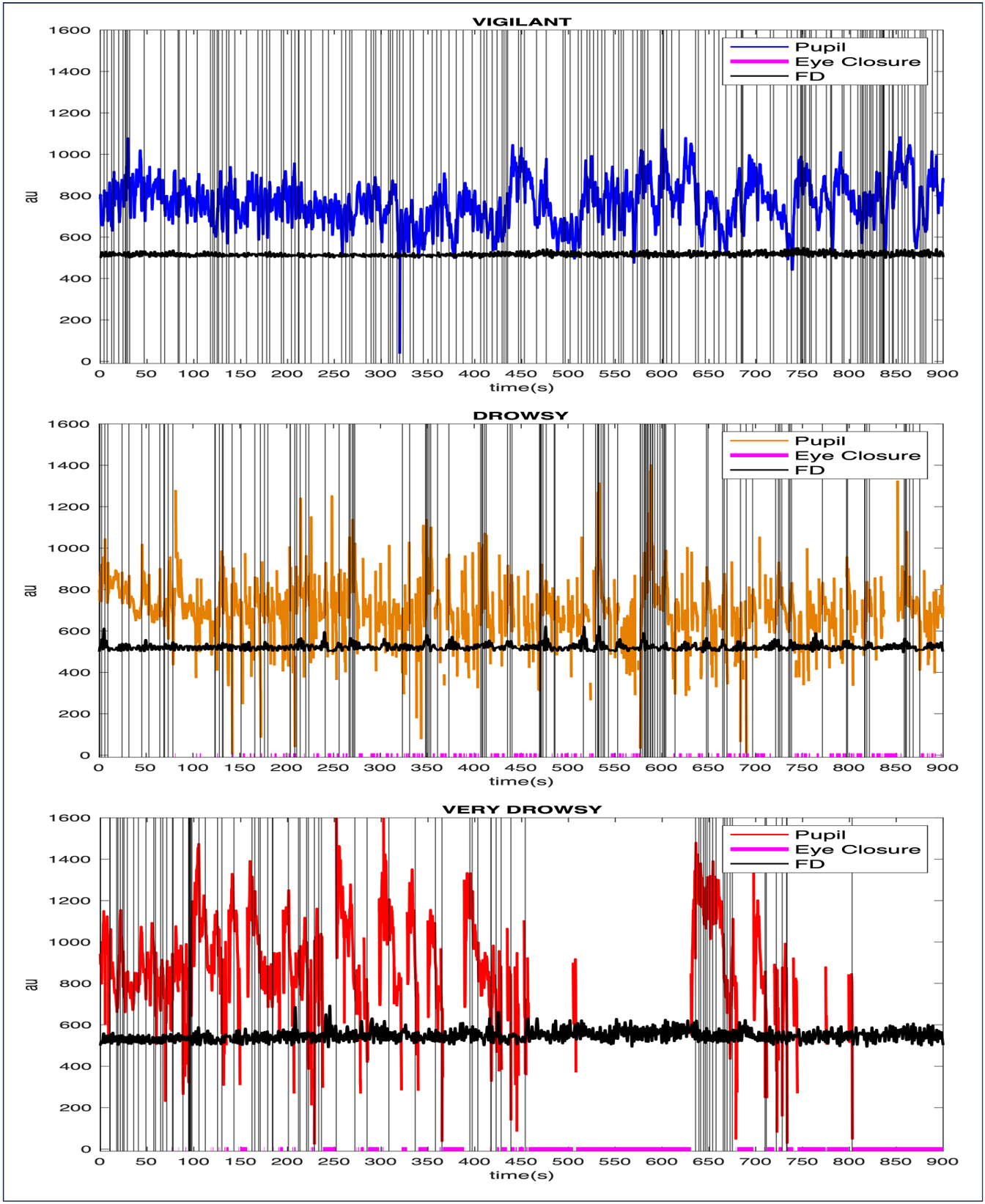


**Supplementary Figure 4**

Coefficient of Variation of BIPR features versus drowsiness (% eye-closure) plots

**
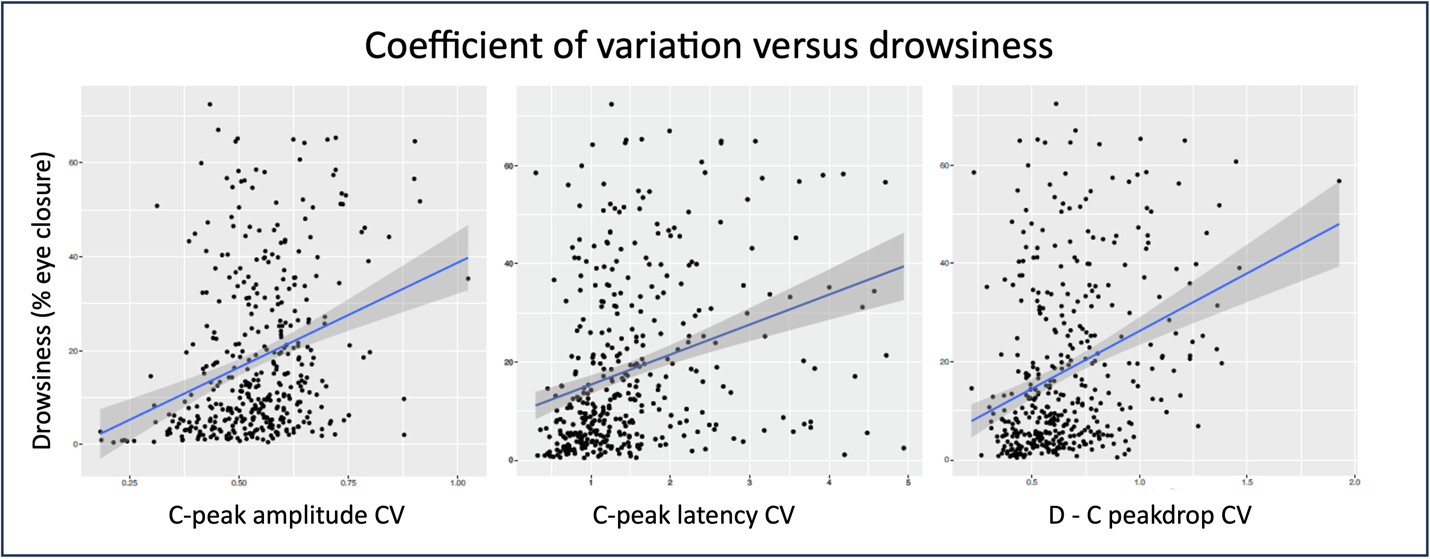
**

**Pre- and Post-eye closure analyses**

We calculated pre-blink pupil size and pupil closure speed (i.e., pupil derivative/change) for the vigilant and drowsy states. Below is an example plot from two different subjects with two runs with the same states (vigilant-vigilant and drowsy-drowsy) plotted from -50ms to blink moment to depict the pre-blink closure behavior (red vertical line is the full closure blink moment, and the very last single point to the right in the plots represents the mean pre-blink pupil size and pupil size derivative (in the window of -50ms to blink moment)). Error bars represent standard deviation across all blink events:


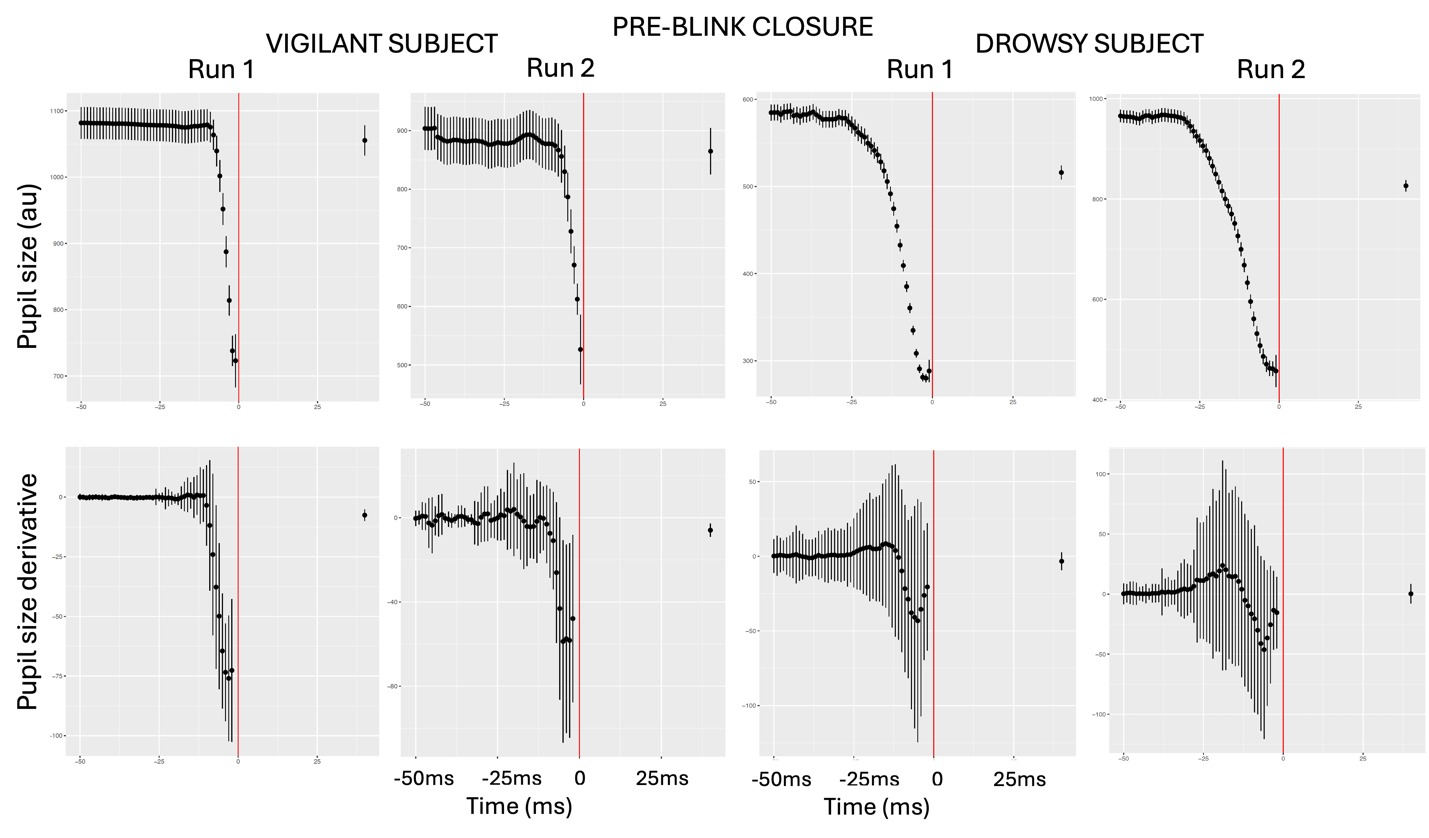


**Figure:** Pre-blink pupil size and pupil closure speed (i.e., pupil derivative/change) for the vigilant and drowsy states plotted from -50ms to blink moment to depict the pre-blink closure behavior. Red vertical line is the full closure blink moment, and the very last single point to the right in the plots represents the mean pre-blink pupil size and pupil size derivative (in the window of -50ms to blink moment). Error bars represent standard deviation across all blink events.

As can be seen in the plots, major pupil size decrements occurred in the last ~20ms for both vigilant and drowsy states but it the drop to achieve full closure is sharper in the vigilant than the drowsy state.

Next, we analyzed pupil size and its derivative/changes in three smaller windows (-60ms to -40m ; -40ms to -20ms; -20ms to 0ms) before blinks with ANOVA approach:


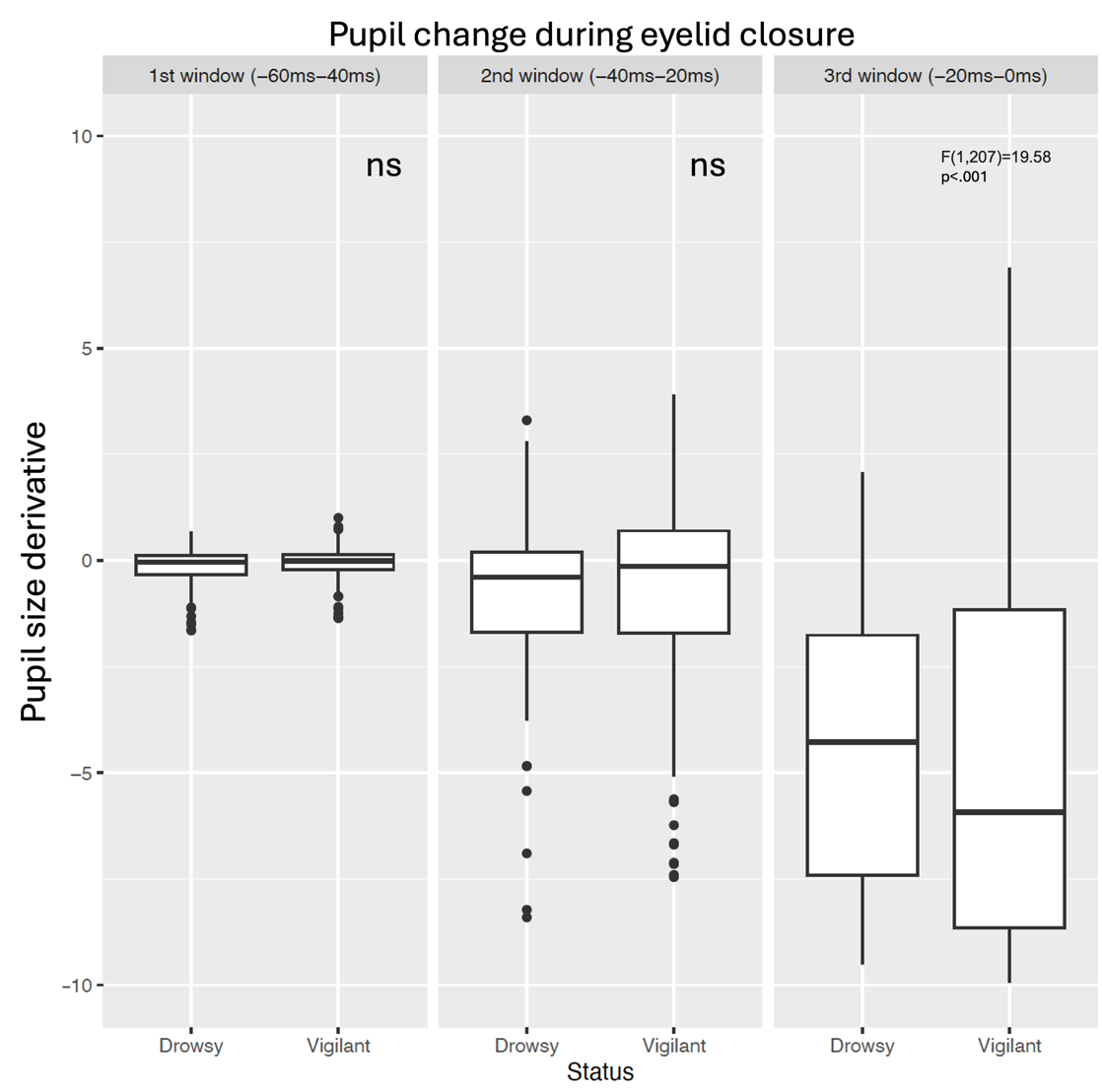


**Figure**: Pre-blink pupil derivative/changes.

Pupil size derivative showed main effects of State (F(1,621)=14.46, p<.001) and Interval (F(2,621)=329, p<.0001) and an interaction effect between Status and Interval (F(2,621)=16.02, p<.001). Resolving this interaction showed that there was a significant difference between states only for the 3^rd^ window (-20ms to 0ms) (F(1,207)=19.58, p<.001).


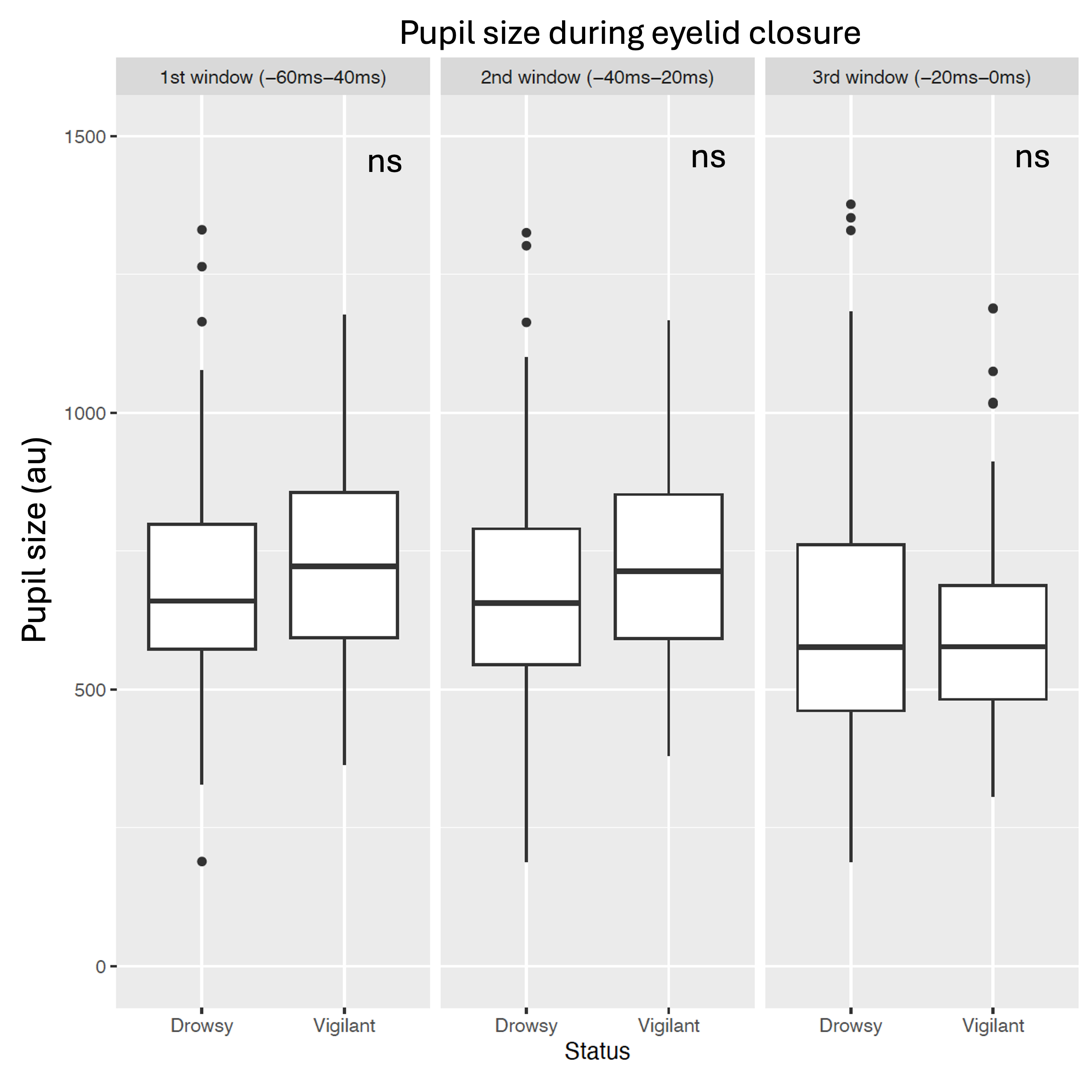


**Figure**: Pre-blink pupil size changes.

On the other hand, pupil size did not differ between the vigilance states but differed only between the time windows (F(2,621)=14.22, p<.001).

Thus, there was no significant difference between the pre-blink pupil sizes. While there was a significantly slower (smaller) change/derivative in the drowsy state -only in a short time interval (~20ms) just before the blink-, such a closure-speed related pupil difference did not create a significant effect in the baseline pupil-size calculations (probably due to the fact that vigilant state has a slightly larger pre-blink pupil size, and together with the faster drop in size for the vigilant state and the slower drop in the drowsy state, comparing the mean area/size of the pupil between the states resulted in comparable sizes).

We did the same analysis for the eye-lid opening (end of blink closure). We calculated post-blink pupil size and eye-lid opening pupil change (i.e., pupil derivative) for the vigilant and drowsy states. Below is an example plot from two different subjects with two runs with the same states (vigilant-vigilant and drowsy-drowsy) plotted from 0ms (end of eye closure) to 300ms to depict the post-blink eyelid opening behavior (red vertical line is the end of the full pupil closure blink moment, and the very first single point to the left in the plots represents the mean post-blink pupil size and pupil size derivative (in the window of 0ms to 100ms). Error bars represent standard deviation across all blink events:


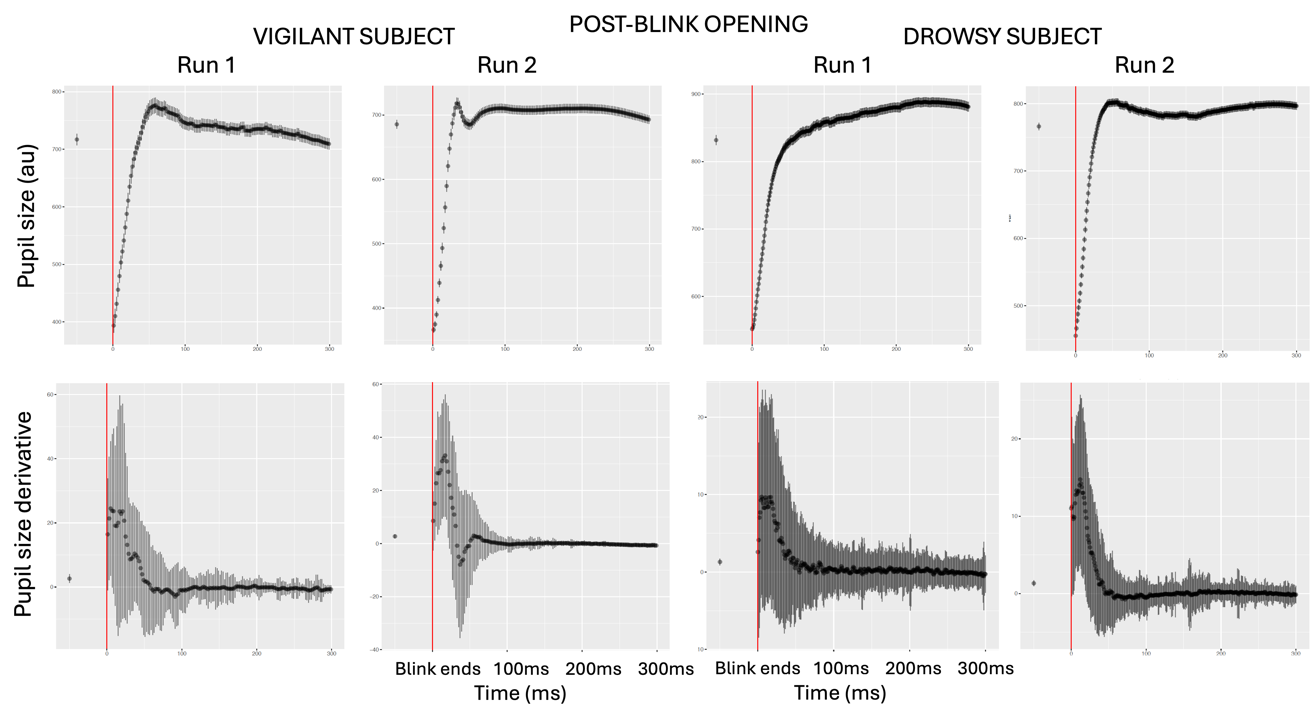


Pupil size derivative showed main effects of State (F(1,621)=16.45, p<.001) and Interval (F(2,621)=197, p<.0001and an interaction between Status and Interval (F(2,621)=10.62, p<.001). Resolving this interaction showed that there was a significant difference between state only for the 1st window (0ms to 40ms) (F(1,207)=16.8, p<.001).


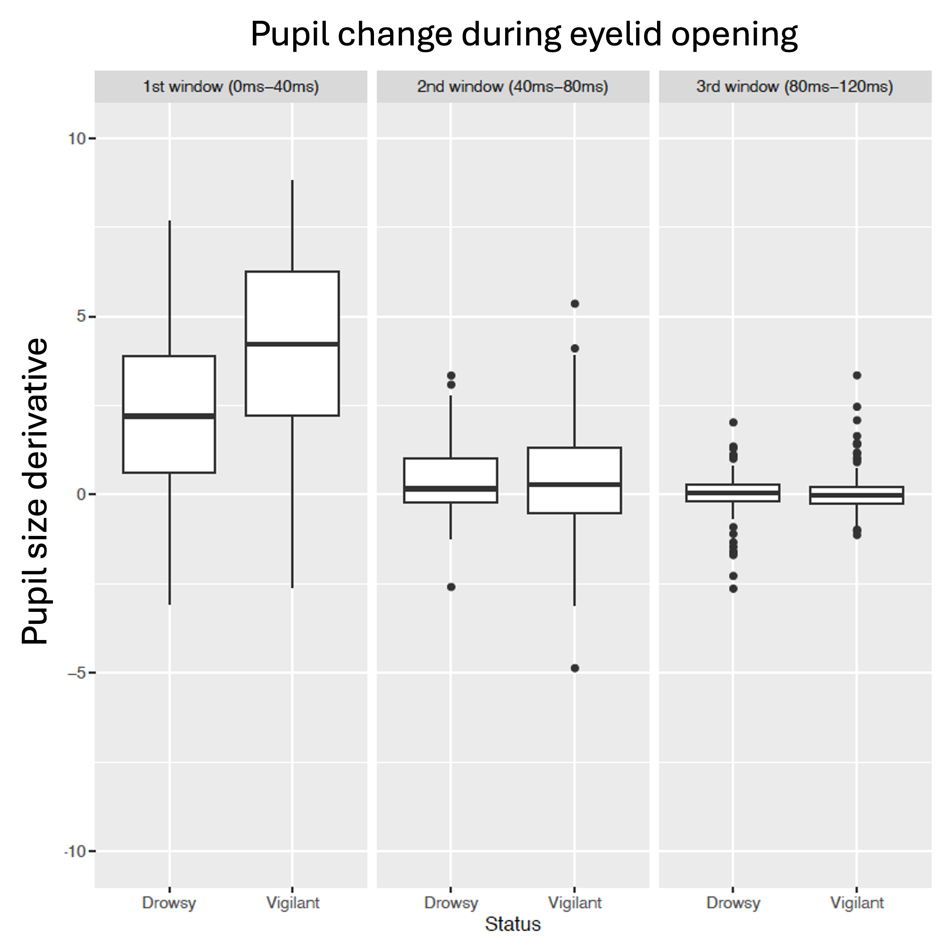


On the other hand, pupil size did not differ between the vigilance states but differed only between the time windows (F(2,621)=13.58, p<.001).


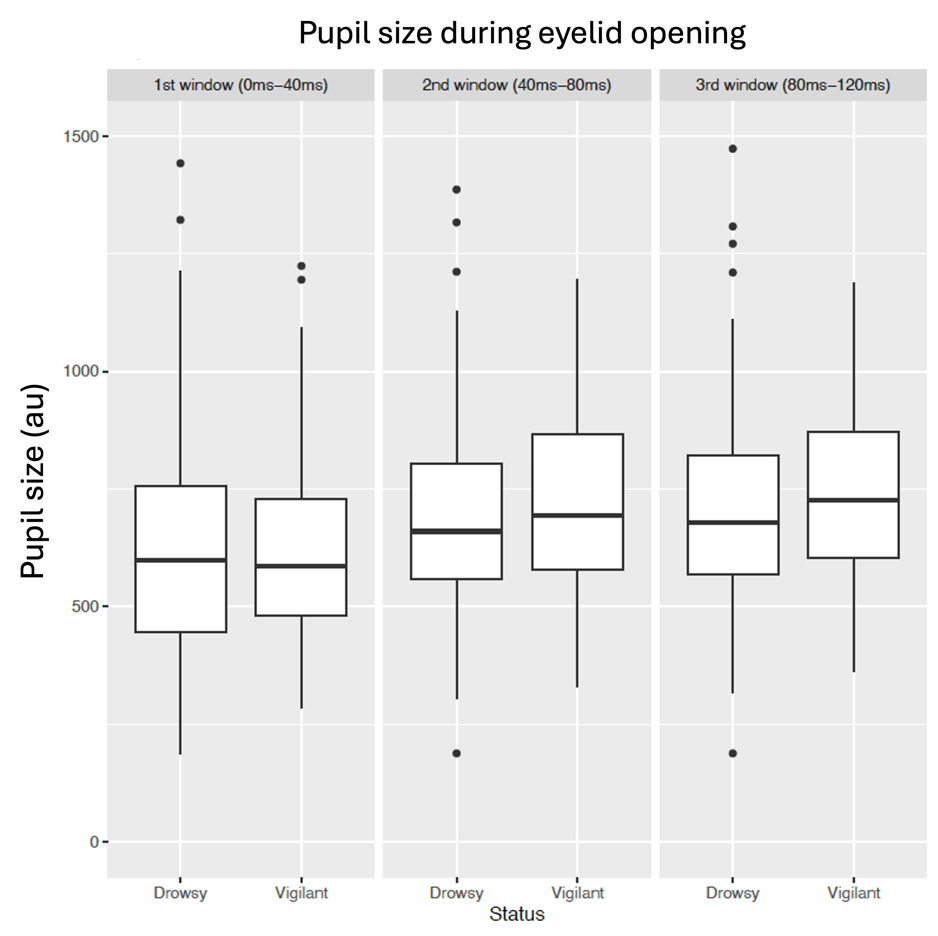


Thus, there were no significant differences between the post-closure pupil sizes across states. There was a faster size increment in the vigilance state only within a short time interval (~40ms).

Thus, partial eye-lid closures are unlikely to confound our pupil analyses.

**Root Mean Square (RMS) and data loss**

We calculated mean RMS and mean eye closure/data loss of the pupil for the two states:

RMS:

Vigilant: 731.24

Drowsy: 724.27

Two states were not found to be significantly different from each other via 2-samples t-test (ns., t=.714)

Naturally, the two states had different mean eye closure/data loss as this was our categorization constraint:

Vigilant: %2.03

Drowsy: %31.23
